# Supplementary material for: Chromothripsis during telomere crisis is independent of NHEJ, and consistent with a replicative origin
Source: Genome Res. 2019 May;29(5):737–49. doi: 10.1101/gr.240705.118 (PMC6499312; doi:10.1101/gr.240705.118)
Supplement: Supplemental Material [file supp_gr.240705.118_Supplemental_file_1.zip › contigs/annotated_contigs/DB54/contig.2.DB54_length_424_mean_cov_6.72169811321.docx]

**DB54_length_424_mean_cov_6.72169811321**

```
TCTGCAGTTGCCTGAAGGGCTTTCTAAAATCCACCAGTTTTTGTTCCTACAGTTCCAGATGTGTTTTAGACAAAACCTTTCCCATTCTT
 >chr18:59652052-59652165 + E=2e-56
CCCAGTCATGATGGAAAGAGTCA|G|TTTAAGTATTCCTACACAGCCCTCAGGAGGGGAGAAGGATGATGGGGGAGGTGGATGGAAATC
                        >chr6:8136634-8136946 - E=2e-173
AAGTTATTTATTTAAGGATTATGATCTGAAATCCATGTTTAGAATATATATATTTTTTACCTCAAAGCACTGATCCCAGTTAAAATGCA

AACATTTCAGAAGGGTTGCACTTCAAGACTTTTATTAGTCATTTACCACTTAGTATAAGCTAGCTCCCAAAAGCAAAATTTGAAAACAC

AAATGGGCCTGACACAGTGGCTCGTGCTTGTAATGCCAGCACTTTGGGAGGCTGAGGCAGGCAGATCACT
```
